# Environmentally Friendly Microemulsions of Essential Oils of *Artemisia annua* and *Salvia fruticosa* to Protect Crops against *Fusarium verticillioides*

**DOI:** 10.3390/nano14211715

**Published:** 2024-10-27

**Authors:** Lucia Grifoni, Cristiana Sacco, Rosa Donato, Spyros Tziakas, Ekaterina-Michaela Tomou, Helen Skaltsa, Giulia Vanti, Maria Camilla Bergonzi, Anna Rita Bilia

**Affiliations:** 1 Department of Chemistry Ugo Schiff, University of Florence, via Ugo Schiff 6, Sesto Fiorentino, 50019 Florence, Italy; lucia.grifoni@unifi.it (L.G.); giulia.vanti@unifi.it (G.V.); mc.bergonzi@unifi.it (M.C.B.); 2 Department of Health Sciences, University of Florence, Viale G.B. Morgagni, 50134 Florence, Italy; cristiana.sacco@unifi.it (C.S.); rosa.donato@unifi.it (R.D.); 3 Section of Pharmacognosy and Chemistry of Natural Products, Department of Pharmacy, School of Health Sciences, National & Kapodistrian University of Athens, Panepistimiopolis, Zografou, 15771 Athens, Greece; tziakas.spyridon9@gmail.com (S.T.); ktomou@pharm.uoa.gr (E.-M.T.); skaltsa@pharm.uoa.gr (H.S.)

**Keywords:** microemulsions, essential oils, *Artemisia annua*, *Salvia fruticosa*, GC–MS analysis, phase diagram, dynamic light scattering, *Fusarium verticillioides*, log-5 and log-4 reduction

## Abstract

Essential oils (EOs) are reported to be natural pesticides, but their use to protect crops is very limited due to EOs’ high instability and great volatility. Nanovectors represent a very smart alternative, and in this study, EOs from *Artemisia annua* (AEO) and *Salvia fruticosa* (SEO) were formulated into microemulsions and tested against *Fusarium verticillioides*. The EOs were extracted by steam distillation and analyzed by GC–MS. The main constituents of AEO were camphor, artemisia ketone, and 1,8-cineole; the main constituents of SEO were 1,8-cineole, camphor, α-pinene, and β-pinene. Artemisia ketone and 1,8-cineole were used to calculate the recovery and chemical stability of the microemulsions. The microemulsions were loaded with 10 mg/mL of EOs, and the recoveries were 99.8% and 99.6% for AEO and SEO, respectively. The sizes of the lipid phases were 255.3 ± 0.6 nm and 323.7 ± 2.3 nm for the AEO and SEO microemulsions, respectively. Activity against *F. verticillioides* was tested using amphotericin B as the positive control. *F. verticillioides* was very susceptible to both EOs. When loaded in the microemulsions, AEO and SEO remained very active at a dose of 1.4 and 1.2 mg, with a 99.99% reduction of *F. verticillioides*. The findings suggest AEO and SEO microemulsions are suitable carriers for the protection of crops against *F. verticillioides*.

## 1. Introduction

The continuous increase in the world population—demographic projections for 2050 indicate that the world population could reach nine billion—has generated the need for agricultural products in sufficient quantities to satisfy the enormous food demand [1]. This has resulted in a significant increase in the use of chemical pesticides to combat crop diseases, but in recent years, there has been a growing awareness among many consumers who are concerned about the side effects of chemical pesticides on human health and the environment [2]. Indeed, pesticide residues have become the focus of attention of European bodies responsible for food safety. In May 2020, the European Commission developed directives linked to the reduction of the use of pesticides with two goals: decrease the use and risk of chemical pesticides by 50% by 2030 and reduce the use of the most dangerous pesticides by 50% by 2030 [3]. These directives are currently under discussion and are awaiting approval, together with the adoption of the new NAP (National Action Plan for the Sustainable Use of Plant Protection Products, last drawn up in 2014) and the issuing of a specific national law on multi-residues, which, based on current scientific evidence, prohibits the co-presence of active ingredients. Hence, from a 2023 study carried out in Italy, 6085 samples of plant and animal origin were analyzed for their content of pesticides; 23.54% of the investigated samples presented multi-residues of pesticides, raising an alarm on the possible additive and synergistic effects of these products on the human organism [4].

In light of the above, the search for naturally derived and low-toxic organic molecules becomes imperative in order to provide an alternative and/or integration to traditional pesticides. The search for alternative solutions to protect crops has become urgent and has recently attracted widespread attention from researchers around the world [1,5,6,7,8,9,10].

Natural products represent a treasure chest of molecules with potent activities, in particular essential oils (EOs). Souihi and coworkers [8] studied the antifungal properties of EOs of various *Eucalyptus* species and successfully evidenced the greater efficacy of these essential oils against four phytopathogenic fungi belonging to the *Fusarium* genus when compared to glyphosate, making tangible their future use in formulations of bio-pesticides. It is worth noting that the herbicide glyphosate, against which there is an ongoing “Glyphosate free” campaign [3], is listed (Group 2A) by IARC as a probable carcinogen as it generates oxidative stress and genetic damage in vitro.

A recent review [7] focused on how several EOs can be useful in protecting tropical fruits such as banana, papaya, mango, and guava. Indeed, EOs have proven to prevent fungal and bacterial infections, as well as to monitor crop infection at both the pre- and post-harvest phases, demonstrating that they are safe and ecologically friendly biopesticides. Very recently, cinnamon EO was reported to possess strong activity in inhibiting the production of mycotoxins such as aflatoxin B1 and fumonisin B1, produced by *Aspergillus flavus* and *Fusarium proliferatum*, respectively [10].

Additionally, some studies investigated the activity of fennel EO loaded in a nanoemulsion against *Fusarium oxysporum*. The nanoformulation was proven to potently inhibit *F. oxysporum*, which causes *Panax notoginseng* root-rot disease [9].

In the current study, the EOs of *Artemisia annua* L. (AEO) and *Salvia fruticosa* Mill. (SEO) were selected to verify their antifungal properties in order to establish a theoretical basis for their future use as potential pesticides, thanks to the numerous studies reporting on their antimicrobial activities [5,11,12,13,14,15].

In particular, the strong antifungal properties of *A. annua* essential oil against *Fusarium oxysporum* and *Fusarium solani*, associated with *Panax notoginseng* root-rot disease, were assessed [5]. Furthermore, *S. fruticosa* essential oil also showed antifungal activity against *Fusarium* spp., principally *F. solani* f. sp. *cucurbitae* [15].

Our research aimed to develop nanoformulations loaded with the selected EOs to evaluate the activity on a strain of *Fusarium verticilloides*, which, in addition to being a phytopathogen, is also a species capable of producing mycotoxins such as fumonisin, which are dangerous for human health [16].

Encapsulation of EOs in nanodelivery systems was investigated to obtain a nanoformulation able to reduce the volatility of the constituents and increase both water solubility and stability because the essential oils are not stable in the presence of light, oxygen, heat, and humidity, giving processes of oxidation, isomerization, cyclization, or dehydrogenation. Furthermore, the nanocarriers are reported to modulate the release of the loaded molecules and prolong their efficacy, optimizing an adequate use [9,12,17,18].

In the present work, the essential oils and the novel microemulsion based on components with trivial toxicity, widely used for oral administration, were developed. Hence, the microemulsion loaded with the EOs was settled as a low-impact, environmentally friendly nanodelivery system to treat crops against *F. verticillioides.* Microemulsions are transparent, thermodynamically stable nanoformulations consisting of an oily phase, water, surfactants, and cosurfactants. They differ from nanoemulsions, which are not thermodynamically (but only kinetically) stable. They are obtained by spontaneous emulsification and hydrodynamic diameter of droplets up to 100 nm, which result in a large surface area when compared with emulsions. Microemulsions are highly versatile nanoformulations, as reflected by their extensive applications in numerous areas including drug delivery, cosmetics, food science, lubricants, coatings, detergents, antibacterials, and pesticides [19].

Indeed, microemulsions represent one of the most investigated nanovectors to formulate EOs related to food sciences and agriculture applications. Hence, it is easy to prepare the formulation due to the high stability, high safety, and low costs of manufacturing [20].

Within the study, a microemulsion with low surfactant content was selected using a pseudo-ternary phase diagram. The microemulsion was loaded with two essential oils (EOs) and characterized chemically and physically. Finally, the formulations were tested for their antifungal activities, making a comparison between the performances of the pure EOs with those of the microemulsion formulations.

## 2. Materials and Methods

### 2.1. Reagents

Anhydrous sodium sulphate, Tween 80, formic acid, artemisia ketone (3,3,6-trimethylhepta-1,5-dien-4-one, purity 96% by GC), and 1,8 cineole (eucalyptol, 1,3,3-trimethyl-2-oxabicyclo [2.2.2]octane, purity 99% by GC) were from Sigma–Aldrich (Milan, Italy). Vitamin E acetate was purchased from ACEF (Fiorenzuola D’Arda, Piacenza, Italy). Cremophor RH40, GC-grade *n*-pentane, and HPLC-grade acetonitrile were purchased from Merck (Rome, Italy). Labrasol ALF was a gift from Gattefossé (Saint Priest, France). All reagents were used as received without further purification. Distilled water was obtained using a Milli-Q Advantage A10 system (Merck Millipore, Darmstadt, Germany). Amphotericin B was from Thermofisher Diagnostics SpA, Milano, Italy. Sabouraud dextrose broth (SDB) and Sabouraud dextrose agar (SDA) were from Oxoid, Thermo Scientific Diagnostics, Rodano, Milan, Italy.

### 2.2. Plant Material, EOs Extraction, and GC–MS Analysis

The flowering tops of *Artemisia annua* L. were collected in October 2022 in Sesto Fiorentino (Florence, Italy). A voucher specimen (Bilia AA 2022) was identified by Anna Rita Bilia, and it is deposited at the Department of Chemistry (DICUS) in Florence. The flowering aerial parts of *Salvia fruticosa* Mill. were collected in July 2022 from the island of Symi island (SE Aegean, Greece). A voucher specimen (Skaltsa & Goula 007) was identified by Dr. Katerina Goula (Section of Ecology and Systematics, Department of Biology, National and Kapodistrian University of Athens), and it is deposited in the Herbarium of the Department of Pharmacognosy and Chemistry of Natural Products (NKUA) in Athens.

EOs of *A. annua* (AEO) and *S. fruticosa* (SEO) were obtained by hydrodistillation in a modified Clevenger apparatus for 3 h, according to the Hellenic Pharmacopoeia. Hydrodistillation of *A. annua* was performed on fresh comminuted plant material (800 g). Hydrodistillation of *S. fruticosa* (20 g) was carried out with plant material comminuted and air-dried at room temperature for 10 days. EOs were stored in dark sealed vials at 4 °C prior to GC–MS analysis and maintained at −21 °C for the antifungal assays. The density of both the EOs was found to be very close to 1 mg/μL. Voucher samples of AEO and SEO were kept at −21 °C at the Department of Chemistry of the University of Florence under the authentication numbers AEO 11/2022 and SEO 06/2022. *n*-Pentane was used for the collection of the EOs, with the addition of anhydrous sodium sulfate to reduce any moisture. The GC–MS analysis of AEO and SEO was carried out using a Hewlett Packard 7820A-5977B MSD system (Palo Alto, CA, USA) operating in EI mode (70 eV) equipped with an HP-5MS fused silica capillary column (30 m × 0.25 mm; film thickness 0.25 µm) and a split-splitless injector. The temperature program for the analysis of AEO was from 50 °C to 290 °C at a rate of 4 °C/min. Helium was used as the carrier gas at a flow rate of 1.0 mL/min. The temperature program for the analysis of SEO was 60 °C at the time of the injection, and then it was raised to 300 °C at a rate of 3 °C/min and subsequently held at 300 °C for 10 min. Helium was used as a carrier gas at a flow rate of 2.0 mL/min. The injected volume of the samples was 1 μL. The analysis was repeated three times. Retention index (RI) values were calculated using a linear equation [21] based on a homologous series of n-alkanes from C9 to C24. The identification of the chemical components was based on a comparison of RI values and mass spectra fragmentation patterns with those reported in the NIST/NBS and Wiley libraries, as well as those described by Adams [22] and other literature data.

### 2.3. Development of Microemulsion

Selection of surfactants and oily phase was based on the solubility of the EOs and chemical characteristics of the excipients. In conclusion, the microemulsion was developed using vitamin E acetate as oil and Cremophor RH 40 and Labrasol ALF as surfactants by using a pseudo-ternary diagram. The chemical composition and some physical characteristics of surfactants are listed in Table 1.

To ascertain the range of compositions within which a microemulsion could be identified, a series of mixtures were prepared, varying the ratio of S_mix_ (a fixed 1:1 mixture of surfactants) to oil (10:1, 9:1, 8:2, 7:3, 6:4, 5:5). The analysis was not extended to other ratios for the purposes of this study, as this would be a topic for further investigation into the study emulsion system and phase separation. The phase diagram of the W–S_mix_–Vitamin E acetate system was built by recording the systems prepared when diluting the original sample of S_mix_ with oil. Visual observation, phase separation, and dynamic light scattering (DLS) were performed to identify the systems as microemulsions, emulsions, or viscous phases (gels or lyotropic liquid crystals). Microemulsion was prepared as previously reported, with some slight modifications. Briefly, the S_mix_ and the oil were mixed homogeneously and placed under gentle stirring at 35 ± 2 °C, and water was added using the *titration* method [26]. To determine the maximum solubility of EOs in the selected microemulsions, a series of tests were conducted, gradually added to the microemulsion system. The process was carefully monitored to identify the point at which the originally clear microemulsion turned into a slightly opalescent emulsion, indicating the saturation limit had been reached. Recovery was obtained using HPLC.

### 2.4. HPLC Analysis to Evaluate the Recovery of the EO

The recovery of AEO and SEO was evaluated and expressed as the percentage of artemisia ketone in AEO and as the percentage of 1,8-cineole in SEO. Artemisia ketone and 1,8-cineole were chosen as standards since they represent main monoterpenes and have high UV absorbance, allowing easy detection. Recovery percentage (R%) was determined by the following Equation (1):

(1) $$Recovery\;\%=\frac{detected\;mg\;}{total\;mg}*100$$

Quantitative analysis of the marker constituents of AEO and SEO were carried out with a HPLC 1200 instrument equipped with a DAD (Agilent Technologies, Santa Clara, CA, USA). A column Kinetex Eclipse, Prestons, NSW, Australia, XDB-C18 (150 mm × 4.6 mm; 5 μm) with a flow rate of 0.5 mL/min at 27 °C was used. A binary mixture of ACN and acid water (pH 3.2 with formic acid) was used for both microemulsions. The UV–Vis spectra were recorded in the 200–600 nm range, and the chromatograms were acquired at λmax = 240 nm. The linearity was determined on six concentration levels of artemisia ketone and 1,8-cineole dissolved in methanol (from 0.2 to 2.5 mg/mL) with three injections for each level. The coefficient of linear correlation was higher than 0.999. An amount of 100 μL of microemulsion was dissolved in 1 mL of methanol before analysis. The following multistep linear solvent gradient was used: 0 min, 60% can; 0.10–15 min, 80% can; 15–20 min, 100% can; 20–25 min, 100% can; 25–30 min, 60% can; and 30–35 min, 60% ACN. For the marker of SEO, the following multistep linear solvent gradient was used: 0 min, 30% can; 0.10–45 min, 100% can; and 46–60 min, 30% ACN.

### 2.5. Characterization of Microemulsions

Empty and EO-loaded microemulsions were evaluated for their particle size and polydispersity index using DLS. Experiments were performed at 25 °C without any further dilution of the samples. All measurements were performed using a Nano ZS Zetasizer (Malvern Instruments Ltd., Malvern, UK) equipped with a He-Ne laser of 532 nm at a scattering angle of θ = 173°. Quartz standard cuvettes were used for size measurements.

### 2.6. Antifungal Activity

The antifungal activity was evaluated using a microdilution method against a *Fusarium verticillioides* GB1 strain. It was isolated from Amaranth flour, and the taxonomical classification was confirmed by sequencing EF1 and ITS-LR. The analysis was performed via the Basic Local Alignment Search Tool (BLAST) to find regions of local similarity between sequences. The results showed 100% similarity for all the genes belonging to *F. verticillioides* [27]. The *Fusarium* strain used in the tests was kept in water at 4 °C in the fridge. Routine steps were taken. The strain was grown in Sabouraud dextrose broth (SDB) and incubated for 5 days at 28 °C. After incubation, the revived strain was grown on Sabouraud dextrose agar (SDA) at 28 °C for 5 days. The cells were harvested by adding 10 mL of sterile distilled water containing 0.05% Tween 80 and scraping the surface of the culture. The fungal concentration was evaluated both by spectrophotometric reading (BioPhotometer Eppendorf srl, Zevenhuizen, The Netherlands) (OD 600) and by counting subcultures on SDA incubated at 28 °C for 5 days. The concentration of the fungal stock solution was between 1 and 1.5 × 10^7^ colony-forming unit (CFU)/mL. In our study, the fungal inocula used were those of standardized testing (in the range 10^5^–10^8^ CFU/mL), significantly higher than those found in most potential end-use settings (e.g., ~10^2^–10^4^) [28]. Scalar amounts of EOs and microemulsions loaded with AEO and SEO (from 180 µL to 100 µL corresponding to 1.8 and 1 mg of EO) were added to the wells to achieve a percentage concentration ranging from 90% to 50% in the SDB culture medium. To all the wells, 20 µL of the fungal stock solution was added. Negative and positive controls were prepared. The antibiotic amphotericin B was included as a positive control, while SDB was the negative control. The empty microemulsion was also tested on the strain in order to exclude their possible antifungal activity, as they contain surfactants. The plate was sealed and incubated for 24 h at 28 °C. Subsequently, the entire contents of the wells (200 µL) were included in Petri dishes using SDA and incubated at 28 °C for 5 days. The test was repeated twice in triplicate. The antifungal activity was evaluated using a microdilution method against *F. verticillioides* strains grown in SDB and incubated for 5 days at 28 °C. After incubation, the revived strain was grown on SDA at 28 °C for 5 days. The capacity of reducing the number of *F. verticilloides* was calculated by log reduction, i.e., 4-log reduction corresponded to 99.99% reduction and 5-log reduction corresponded to 99.999% reduction. For a product to pass EN 1275, it must be able to achieve 4-log reduction against the respective test [29].

Log reduction calculation can be obtained from the following Formula (2):

(2) $$Log\;Reduction={log}_{10}\left(A\right)-{log}_{10}(B)$$

where A is the number of the viable microorganisms before treatment, and B is the number of the viable microorganisms after treatment.

### 2.7. Statistical Analysis

To compare the characteristics of the three microemulsions studied, a one-way analysis of variance (ANOVA) was performed. The ANOVA was applied to determine whether there were significant differences between the group means. The significance level was set at *p* < 0.05. Following the ANOVA, a test was conducted as a post-hoc analysis to identify specific differences between the groups. This test allowed for the comparison of all possible group pairs to determine which combinations exhibited statistically significant differences. All statistical analyses were performed using OriginPro^®^ 2022 software.

## 3. Results and Discussion

### 3.1. Extraction and Composition of the Essential Oils

Hydrodistillation of *A. annua* gave a yield of 0.45% *w*/*w*, and hydrodistillation of *S. fruticosa* gave a yield of 3.6.% *w*/*w*. Composition of the EOs was obtained by GC–MS analysis. Overall, 27 and 75 compounds were identified in AEO and SEO, representing 97.0% and 94.6% of the total components, respectively (Table 2 and Table 3). The main constituents of AEO (Table 2) were camphor (18.1%), 1,8-cineole (16.0%), and artemisia ketone (24.3%). Monoterpenes represent the most abundant group of compounds in the EO, representing over 92.3% of the total composition (Table 4). The oxygenated ones account for up to 80.3% of AEO. Sesquiterpenes are less abundant and are represented by only hydrocarbon ones (4.3%), while no oxygenated sequiterpenes were found, and non-terpenoid derivatives represented 0.4%.

The profile of the AEO’s constituents is very similar to those reported in previous reports, characterized by a high content of monoterpenes and sesquiterpenes, whose main constituents are artemisia ketone (up to 68%), 1,8 cineole (up to 51.5%), and camphor (up to 48%). In the literature, it is reported that constituent profiles are influenced by chemotype or subspecies, geographic area, harvesting season, the pH of the soil, the use of fertilizers, and the extraction method [12]. Composition was particularly similar to that of AEO previously investigated for their antimicrobial activities [12,13].

Regarding the EO of *S. fruticosa*, the main chemical constituents (>5.0%) of SEO were 1,8-cineole (38.5%), camphor (7.8%), α-pinene (7.1%), and β-pinene (6.2%) (Table 3). The EO presented high amounts of oxygenated monoterpenes (65.2%), followed by monoterpene hydrocarbons (19.4%) and oxygenated sesquiterpenes (5.3%) (Table 5). Sesquiterpene hydrocarbons were found in lower amounts (4.5%). Several studies reported the chemical constituents of *S. fruticosa* EO due to its economic importance [15,30,31,32,33,34].

Our results were generally in accordance with previous reports on SEOs indicating 1,8-cineole as the major component. However, there are noticeable variations in terms of the relative percentages of the components, which could probably be attributed to the different geographical areas sampled and the year of the sample collection. Although cis-thujone (4.8%) and trans-thujone (4.6%) were found, the total thujone content was low (less than 10.0% of the total oil), as previously mentioned [31]. In previous works, oxygenated monoterpenes were also the main chemical classes, followed by monoterpene hydrocarbons [15,32]. This study represents the first report on *S. fruticosa* EO collected from Symi island.

### 3.2. Microemulsion Development

Cremophor RH 40 was selected as the surfactant because of the high solubility of EOs in this constituent and because it is a GRAS (generally recognized as safe) substance [35]. The main constituent of this product is glyceryl polyethylene glycol oxystearate, which, together with fatty acid glyceryl polyglyceryl esters, forms the hydrophobic part of the product, aiding in the stabilization of the microemulsion. Labrasol ALF is known for its high solubilizing properties and extensive use as an O/W surfactant for microemulsions.

Vitamin E acetate was chosen as the oily phase due to its lipophilic nature and great ability to solubilize the EOs, which contribute to the stability of EO-loaded microemulsion.

**Figure 1:**
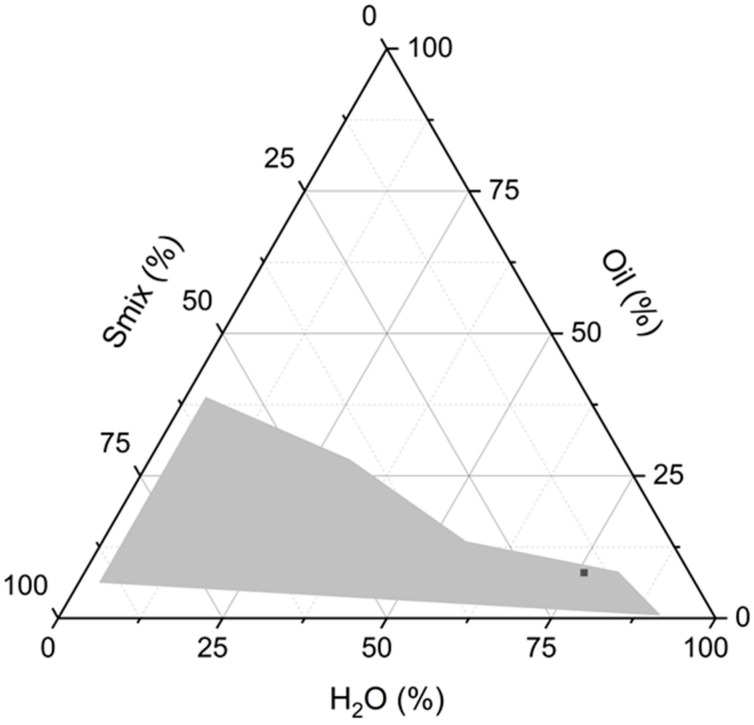
Phase diagram of water–vitamin E acetate–Cremophor RH 40 plus Labrasol ALF (surfactants in a 1:1 ratio) system at 35 °C. The grey area indicates the presence of the microemulsion. The dark grey symbol (▪) identifies the composition of the selected microemulsion.

The O/W microemulsion composition was obtained by pseudo-ternary phase diagram using Cremophor RH 40 and Labrasol ALF as surfactants mixture (S_mix_). The surfactants 1:1 molar ratio was selected from our previous screening experiments as suitable in terms of replacement of more toxic surfactants. The phase diagram of the system was built by diluting different ratios of S_mix_ and oil with water using the titration method. The diagram is reported in Figure 1.

The phase diagram reveals a quite large O/W microemulsion domain, indicating a broad range of compositions where microemulsions can form. Notably, within this region, the content of non-water components in the microemulsion can be reduced to as low as ~20%, demonstrating the system’s ability to accommodate high water content while maintaining stability. The phase diagram was constructed across a surfactant mixture concentration range of up to 90%. However, at these higher concentrations of surfactants, very viscous systems were observed, which are less desirable for practical applications, particularly those requiring ease of use, such as sprayability.

Given the need to develop an ecological and cost-effective microemulsion system with minimal toxicity, the selection of the final composition was carefully guided by specific criteria. Although the microemulsion region in the phase diagram is extensive, only compositions containing more than 70% water were considered for further study. This high water content was targeted to reduce the overall viscosity of the system, facilitating a low-viscosity formulation that could potentially be sprayable. Such a property is crucial for applications that require easy and even distribution, such as in agricultural treatments.

The selected microemulsion composition was designed to maximize the oil content while minimizing the surfactant concentration. This approach was taken to achieve several key objectives. Firstly, reducing the surfactant concentration helps minimize the potential toxicity of the microemulsion. Surfactants, while essential for stabilizing the microemulsion, can have adverse effects on both human health and the environment if used in excessive amounts. Therefore, selecting a formulation with the lowest feasible surfactant content reduces the risk of irritation or toxicity, making the system safer for both ecological and dermal applications.

Secondly, by maximizing the oil content relative to surfactants, the microemulsion ensures that enough of the active ingredients, Vitamin E acetate and essential oils, is present to exert their desired antifungal and antioxidant effects. Moreover, the oil acts as the primary carrier for the active compounds, enhancing their solubility and bioavailability in the target application. This increase in bioavailability is crucial for ensuring the effectiveness of the essential oils in providing their antifungal properties.

Thirdly, the high water content in the microemulsion not only contributes to a reduction in viscosity but also enhances the formulation’s potential to be used as a sprayable product. A less viscous and more fluid system is advantageous for applications requiring an even and efficient distribution over large surface areas. In agricultural settings, for example, a sprayable formulation allows for easier application on plants, providing an even coating that maximizes the antifungal activity of the essential oils.

Lastly, the selection of this microemulsion composition aligns with environmental and economic considerations. A formulation with a lower concentration of surfactants minimizes the environmental impact, as surfactants can pose ecological risks if they accumulate in the environment. Additionally, by reducing the overall surfactant content and using water as a primary component, the formulation becomes more cost-effective. This cost reduction makes the microemulsion an economically viable option for large-scale applications. Furthermore, using water as the main solvent enhances the formulation’s safety profile, ensuring it aligns with green chemistry principles by reducing reliance on potentially hazardous chemicals. In detail, the selected microemulsion was developed using vitamin E acetate as oil (8.2%) and Cremophor RH 40 and Labrasol ALF as surfactants (14.8%) in a 1:1 ratio.

In summary, the selected microemulsion composition achieves an optimal balance between maximizing the oil phase to ensure the effective delivery of active ingredients and minimizing surfactant concentration to reduce toxicity and environmental impact. By incorporating a high water content, the microemulsion achieves the desired low viscosity, enhancing its applicability as a sprayable or easily spreadable formulation. This careful optimization makes the microemulsion an ecological, cost-effective, and less toxic delivery system suitable for antifungal treatments of crops.

### 3.3. Development of Microemulsions Loaded with EOs

Due to their complex composition with numerous functional groups, EOs are general very difficult to solubilize in diluted surfactant solutions. The maximum solubility of EOs in the selected microemulsion was tested by adding to the mixture of oil phase and S_mix_, successively, volumes of the two essential oils until the original microemulsion turned into a slightly opalescent emulsion.

The loading capacity for both SEO and AEO was found to be 10 mg/mL in the microemulsion. This testing was performed at room temperature, where the essential oils were added drop by drop to the formulation under gentle mixing conditions. This gradual addition ensured that the oils were uniformly dispersed within the microemulsion without causing immediate destabilization. The gentle mixing helped maintain the delicate balance of the microemulsion, allowing for the maximum incorporation of the essential oils while preserving the system stability. This method confirmed that the selected microemulsion is capable of effectively solubilizing a significant amount of EOs, making it a suitable carrier for these complex, bioactive components.

The maximum solubility of EOs in the selected O/W microemulsion was tested by adding, successively, volumes of the two essential oils until the original microemulsion turned into a slightly opalescent emulsion. The loading was 10 mg/mL for both EOs, which were added drop by drop to the formulation at room temperature under gentle mixing. Composition of the developed microemulsions loaded with SEO and AES are reported in Table 6.

### 3.4. Microemulsions Characterization

**Figure 2:**
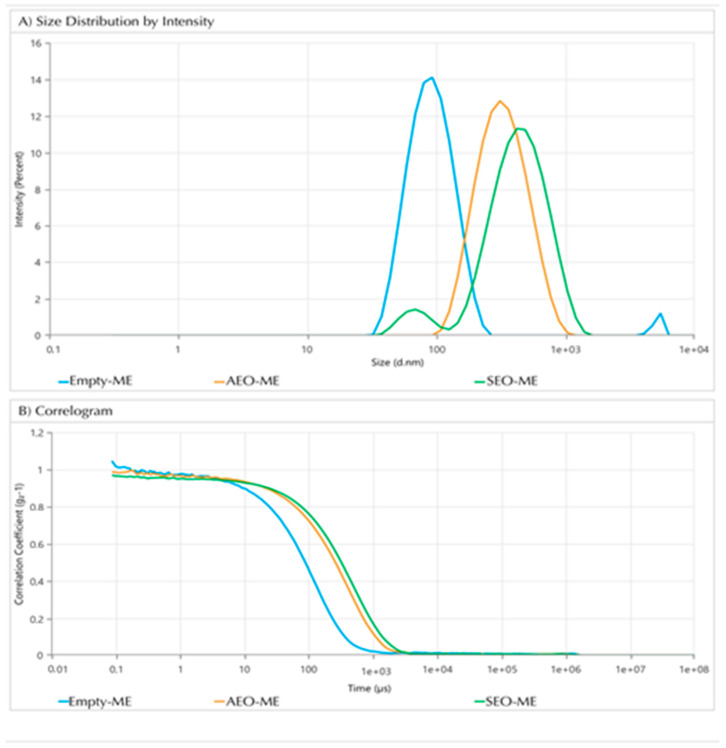
Size and size distribution (**A**) and correlogram (**B**) of empty microemulsion (light blue line) and microemulsions encapsulating SEO (green line) and AEO (orange line).

Characterization of empty microemulsion was carried out by DLS, evidencing globules with a size of about 86 nm and a polydispersity index of about 0.260, as reported in Table 7. The small dimension and low polydispersity index indicated a well-structured and stable system. As expected, the incorporation of the EOs in the microemulsions led to an increase in droplet size because the addition of the EOs increased the volume of the globules (255.3 ± 0.6 nm and 323.7 ± 2.3 nm, respectively, Table 7, Figure 2). Sizes and polydispersities of the loaded microemulsions with EOs were significantly different (*p* < 0.05) when compared to the empty microemulsion.

However, despite this increase, the droplets remained within the nanoscale, which is crucial for maintaining solubility and stability, ensuring an efficient delivery of the EOs (Figure 2).

EOs recoveries in the microemulsions loaded with 10 mg/mL of AEOs and SEOs were obtained by HPLC analysis. The value percentages were very high, 99.8% and 99.6%, respectively.

Indeed, the developed microemulsions loaded with EOs displayed no signs of changes in the visual aspect and in the average size of the oil droplets, and no variation of the EO recovery during refrigerated storage for 4 weeks was observed. Noteworthily, the correlation coefficient over time (µs) provides information on the dynamics of particle movement in the system. In the case of the empty microemulsion, the rapid decay in the correlation function is indicative of a smaller droplet size and less complex internal dynamics, which is consistent with its size of 86.2 nm. In the case of microemulsions loaded with the EOs, the curves exhibit a slight deceleration in decay, indicative the largest particle sizes upon loading with EOs. In addition, the similarity in the decay pattern for AEO- and SEO-loaded microemulsions suggested that the microemulsion systems exhibit analogous dynamic stability.

### 3.5. Antifungal Activity

Microemulsions were chosen as suitable nanodrug delivery systems in order to guarantee enhanced solubility and stability of the EOs together with an efficient release. Our study investigated the activity of EOs of *A. annua* or *S. fruticosa* against *F. verticilloides*, the unloaded microemulsions, and the microemulsions loaded with the two EOs. The promising results are reported in Table 8 and Table 9.

#### 3.5.1. Evaluation of EOs Antifungal Activity

In order to identify EOs with high antifungal activity to be included in the microemulsions, we performed screening tests with five *Salvia* taxa and *A. annua*. We used the microdilution method against *F. verticillioides* strain. All EOs were firstly tested to the amount of 100 mg corresponding to 100 μL. At this concentration, only *S. fruticosa* and *A. annua* showed a potent reduction of *F. verticillioides*, and, therefore, they were used for the successive tests at lower amounts (20, 10, and 2 mg), as reported in Table 8.

#### 3.5.2. Evaluation of Antifungal Activity of Developed Microemulsion and Microemulsions Loaded with AEO and SEO

The empty microemulsion and the microemulsions loaded with AEO and SEO were tested for antifungal activity at a maxima dose of 180 mg (about 180 μL) because the entire contents of the wells were 200 µL, and to each well, 20 µL of the fungal stock solution was added. The antibiotic amphotericin B was included as a positive control, while SDB was the negative control. The plate was sealed and incubated for 24 h at 28 °C. No activity against *F. verticilloides* was found for the empty microemulsions. These findings suggest that both of the non-ionic surfactants present in the formulation do not have a direct activity against the bacterial cytoplasmic membrane or other mechanisms, which can result in bactericidal properties. According to our results, great biocompatibility was found, which resulted in the noticeable safety of these nanodrug delivery systems.

Microemulsions loaded with SEO and AEO were added in the amount of 180 mg and at scalar concentrations to obtain five scalar dosages (1.8, 1.6, 1.2, and 1 mg). The log reductions of *Fusarium* obtained with the microemulsions loaded with SEO and AEO are reported in Table 9.

For the SEO microemulsion a, a 4-log reduction was found at the amounts of 1.8, 1.6, 1.4, and 1.2 mg of SEO loaded in the microemulsion. The microemulsion containing 1 mg of this SEO produced a 3-log reduction and was therefore not considered effective.

The microemulsion loaded with AEO was active (logarithm reduction of 4) up to the amount of 1.4 mg, thus showing a lower antifungal activity with respect to the microemulsion containing SEO.

## 4. Conclusions

In recent years, a significant increase in the use of chemical pesticides to combat crop diseases has seen a growing awareness among many consumers concerned about the side effects of chemical pesticides on human health and the environment. The aim of our research was to contribute to the development of possible formulations to be used in agriculture as bio-pesticides, given that this field of research is still little investigated. In this study, two EOs were selected for their activity against *F. verticilloides*: the EO of *A. annua* (AEO) and the EO of *S. fruticosa* (SEO). The EOs used in this study show a composition similar to the ones reported in the literature using GC–MS analysis. In particular, these two EOs are particularly interesting products for possible application in the control of *F. verticilloides*. AEO represents a valuable by-product that can be obtained from the plant *A. annua*, widely cultivated for the isolation of artemisinin, a non-volatile sesquiterpene endoperoxide with potent antimalarial properties [12]. SEO is extensively cultivated because it has economic importance as a flavoring agent in perfumery and cosmetics, with a high yield (3.6%). Due to the high chemical instability and volatility of EOs, appropriate formulations should be used, and, in particular, nanosized microemulsions are able to protect the volatility of ethereal oils and protect them from degradation in the presence of light, air, and temperature [21].

The proposed microemulsions are environmentally friendly, with a high content of water, minimum surfactant, and oil concentrations encapsulating up to 10% EOs. The microemulsions could represent innovative formulations that can be used in the field. The nanocarriers, which are easily scaled up, are completely biodegradable, and the oily internal phase has antioxidant properties, which contribute to the stability of the component of the formulation, preserving the biological properties of SEO and AEO. 

## Figures and Tables

**Table 1:** Chemical formula and characteristics of selected surfactants.

Surfactant	Chemical Structure	HLB	CMC (mg/L)
Cremophor RH 40	Polyoxyl 40 hydrogenated castor oil	15 [23]	9 × 10^−3^ [24]
Labrasol ALF	Caprylocaproyl Polyoxyl-8 glycerides	12 [25]	42 [25]

CMC: critical micellar concentration.

**Table 2:** Chemical composition of *Artemisia* essential oil (AEO).

No.	RI_L_	RI_C_	Constituents	%
1	921	923	santolina triene	1.1
2	933	931	α-pinene	1.3
3	945	945	camphene	2.2
4	958	960	sabinene	2.1
5	974	972	β-pinene	0.6
6	988	989	myrcene	4.1
7	995	993	yomogi alcohol	1.9
8	1013	1011	α-terpinene	0.6
9	1025	1023	1,8-cineole	16.0
10	1060	1058	artemisia ketone	24.3
11	1068	1066	*cis*-sabinene hydrate	0.1
12	1074	1074	artemisia alcohol	7.0
13	1120	1119	dehydrosabinaketone	0.1
14	1136	1136	*trans*-pinocarveol	0.3
15	1140	1141	camphor	18.1
16	1156	1155	β-pinene oxide	5.7
17	1160	1161	pinocarvone	0.9
18	1163	1164	borneol	0.9
19	1175	1174	4-terpineol	1.5
20	1184	1186	α-terpineol	1.1
21	1190	1188	myrtenol	0.6
22	1238	1235	hexyl isovalerate	0.4
23	1372	1372	α-copaene	0.4
24	1411	1410	β-caryophyllene	1.8
25	1457	1456	*(E)*-β-farnesene	0.2
26	1477	1478	germacrene D	1.8
27	1491	1490	bicyclogermacrene	0.1
			Total identified	97.0

RI_L_ = literature retention indices; RI_C_ = calculated retention indices using an n-alkane standard solution C9–C24 in HP-5 MS column.

**Table 3:** Chemical composition of *Salvia* essential oil (SEO).

No.	RI_L_	RI_C_	Constituents	%
1	924	923	α-thujene	0.4
2	932	930	α-pinene	7.1
3	946	945	camphene	1.3
4	974	972	β-pinene	6.2
5	988	987	myrcene	3.6
6	1002	1003	α-phellandrene	0.1
7	1014	1013	α-terpinene	0.1
8	1026	1028	1,8-cineole	38.5
9	1054	1053	γ-terpinene	0.4
10	1065	1062	*cis*-sabinene hydrate	0.2
11	1086	1087	terpinolene	0.2
12	1101	1103	*cis*-thujone	4.8
13	1112	1111	*trans*-thujone	4.6
14	1141	1140	camphor	7.8
15	1158	1156	*trans*-pinocamphone	0.9
16	1162	1160	δ-terpineol	0.2
17	1172	1170	*cis*-pinocamphone	0.2
18	1174	1172	terpinen-4-ol	1.3
19	1179	1178	p-cymen-8-ol	0.1
20	1186	1183	α-terpineol	1.8
21	1194	1191	myrtenol	0.2
22	1207	1212	*trans*-piperitol	0.1
23	1215	1216	*trans*-carveol	0.1
24	1227	1225	nerol	0.2
25	1235	1235	neral	0.1
26	1249	1250	geraniol	0.2
27	1254	1253	linalool acetate	0.3
28	1264	1264	geranial	0.1
29	1283	1279	isobornyl acetate	0.1
30	1289	1290	thymol	0.1
31	1289	1293	*trans*-sabinyl acetate	0.1
32	1298	1296	carvacrol	0.4
33	1316	1314	δ-terpinyl acetate	0.1
34	1346	1348	α-terpinyl acetate	2.4
35	1359	1360	neryl acetate	0.1
36	1374	1375	α-copaene	0.1
37	1379	1378	geranyl acetate	0.1
38	1387	1386	β-bourbonene	0.1
39	1417	1415	(*E*)-caryophyllene	2.9
40	1439	1438	aromadendrene	0.1
41	1452	1449	α-humulene	0.7
42	1478	1475	γ-muurolene	0.1
43	1496	1494	valencene	0.1
44	1513	1510	γ-cadinene	0.1
45	1521	1520	*trans*-calamenene	0.1
46	1522	1523	δ-cadinene	0.2
47	1582	1580	caryophyllene oxide	2.4
48	1592	1590	viridiflorol	0.2
49	1608	1605	humulene epoxide II	0.7
50	1639	1636	caryophylla-4(12),8(13)-dien-5α-ol/or caryophylla-4(12),8(13)-dien-5β-ol	0.6
51	1666	1662	14-hydroxy-(*Z*)-caryophyllene	1.1
52	1668	1667	14-hydroxy-9-epi-(*E*)-caryophyllene	0.2
53	1685	1683	germacra-4(15),5,10(14)-trien-1α-ol	0.1
54	2056	2052	manool	0.3
			Total	94.6

RI_L_ = literature retention indices according to Adams [22]; RIc = calculated retention indices using an n-alkane standard solution C9–C24 in HP-5 MS column.

**Table 4:** Chemical composition of *Artemisia* essential oil (AEO) according to grouped constituents.

Grouped Constituents	%
monoterpene hydrocarbons	12.0
oxygenated monoterpenes	80.3
sesquiterpene hydrocarbons	4.3
oxygenated sesquiterpenes	0.0
non-terpenoid derivatives	0.4

**Table 5:** Chemical composition of *Salvia* essential oil (SEO) according to grouped constituents.

Grouped Comstituents	%
Monoterpene Hydrocarbons	19.4
Oxygenated Monoterpenes	65.2
Sesquiterpene Hydrocarbons	4.5
Oxygenated Sesquiterpenes	5.3
Oxygenated Diterpenes	0.3

**Table 6:** Composition of the microemulsions (ME) selected for antifungal evaluation.

ME	Vitamin E Acetate (% *w*/*w*)	Labrasol ALF (% *w*/*w*)	Cremophor RH 40 (% *w*/*w*)	Water (% *w*/*w*)	AEO (% *w*/*w*)	SEO (% *w*/*w*)
AEO ME	8.2	7.4	7.4	76.0	1.0	
SEO ME	8.2	7.4	7.4	76.0		1.0

AEO: Artemisia essential oil; SEO: Salvia essential oil.

**Table 7:** Size of the oily phase and polydispersity of the microemulsions (ME).

	Empty-ME	AEO-ME	SEO-ME
Size (nm)	86.2 ± 1.3	255.3 ± 0.6	323.7 ± 2.3
Polydispersity Index	0.256 ± 0.009	0.314 ± 0.003	0.305 ± 0.004

AEO: Artemisia essential oil; SEO: Salvia essential oil. All the reported values are statistically significant (*p* < 0.05).

**Table 8:** Logarithmic reductions of *F. verticilloides* subsequently to different concentrations of AEO and SEO.

*F. verticilloides* (CFU/20 µL)	AEO			
2.8 × 10^5^	100 mg	20 mg	10 mg	2 mg
Log reduction	5	5	5	5
	**SEO**			
2.8 × 10^5^	100 mg	20 mg	10 mg	2 mg
Log reduction	5	5	5	4

*F. verticilloides*. 2.8 × 10^7^ CFU/mL. CFU = colony-forming unit. AEO: Artemisia essential oil; SEO: Salvia essential oil. The results are the average of two tests repeated in triplicate.

**Table 9:** Logarithmic reduction of *F. verticilloides* by the microemulsions loaded with AEO and SEO.

*Fusarium* spp. (CFU/20 µL)	AEO Microemulsion				
2.4 × 10^5^	1.8 mg	1.6 mg	1.4 mg	1.2 mg	1.0 mg
Log reduction	4	4	4	3	3
	**SEO Microemulsion**				
2.4 × 10^5^	1.8 mg	1.6 mg	1.4 mg	1.2 mg	1.0 mg
Log reduction	4	4	4	4	3

*Fusarium* spp. 1.2 × 10^7^ CFU/mL. CFU = colony-forming unit. AEO: Artemisia essential oil; SEO: Salvia essential oil. The results are the average of two tests repeated in triplicate.

## Data Availability

Data are contained within the article.

## References

[1] Lahlali R., Ezrari S., Radouane N., Kenfaoui J., Esmaeel Q., El Hamss H., Belabess Z., Barka E.A. (2022). Biological Control of Plant Pathogens: A Global Perspective. Microorganisms.

[2] Ahmad M.F., Ahmad F.A., Alsayegh A.A., Zeyaullah M., AlShahrani A.M., Muzammil K., Saati A.A., Wahab S., Elbendary E.Y., Kambal N. (2024). Pesticides impacts on human health and the environment with their mechanisms of action and possible countermeasures. Heliyon.

[3] European Union, Farm to Fork Strategy. https://food.ec.europa.eu/horizontal-topics/farm-fork-strategy_en.

[4] Legaambiente Agricoltura. https://agricoltura.legambiente.it/2024/05/legambiente-in-marcia-contro-i-pesticidi-stop-alle-sostanze-chimiche-per-un-futuro-piu-sano/.

[5] Ma Y.N., Chen C.J., Li Q.Q., Xu F.R., Cheng Y.X., Dong X. (2019). Monitoring antifungal agents of *Artemisia annua* against *Fusarium oxysporum* and *Fusarium solani*, associated with *Panax notoginseng* root-rot disease. Molecules.

[6] Li S., Wu Q., Sun Q., Coffin S., Gui W., Zhu G. (2019). Parental exposure to tebuconazole causes thyroid endocrine disruption in zebrafish and developmental toxicity in offspring. Aquat. Toxicol..

[7] Mohd Israfi N.A., Mohd Ali M.I.A., Manickam S., Sun X., Goh B.H., Tang S.Y., Ismail N., Abdull Razis A.F., Ch’ng S.E., Chan K.W. (2022). Essential oils and plant extracts for tropical fruits protection: From farm to table. Front. Plant Sci..

[8] Souihi M., Kouki H., Amri I., Maalej I., Souissi A., Trabelsi I., Dhaouadi F., Hamrouni L., Mabrouk Y. (2024). Valorisation of essential oil of *Eucalyptus populifolia* Desf, *Eucalyptus woollsiana* and *Eucalyptus exserta* for agro-industrial purposes. Int. J. Environ. Health Res..

[9] Nie H., Liao H., Wen J., Ling C., Zhang L., Xu F., Dong X. (2024). *Foeniculum vulgare* essential oil nanoemulsion inhibits *Fusarium oxysporum* causing *Panax notoginseng* root-rot disease. J. Ginseng Res..

[10] Phan L.T.K., Le A.T.H., Hoang N.T.N., Debonne E., De Saeger S., Eeckhout M., Jacxsens L. (2024). Evaluation of the efficacy of cinnamon oil on *Aspergillus flavus* and *Fusarium proliferatum* growth and mycotoxin production on paddy and polished rice: Towards a mitigation strategy. Int. J. Food Microbiol..

[11] Chebbac K., Benziane Ouaritini Z., El Moussaoui A., Chalkha M., Lafraxo S., Bin Jardan Y.A., Nafidi H.A., Bourhia M., Guemmouh R. (2023). Antimicrobial and Antioxidant Properties of Chemically Analyzed Essential Oil of *Artemisia annua* L. (Asteraceae) Native to Mediterranean Area. Life.

[12] Risaliti L., Pini G., Ascrizzi R., Donato R., Sacco C., Bergonzi M.C., Salvatici M.C., Bilia A.R. (2020). *Artemisia annua* essential oil extraction, characterization, and incorporation in nanoliposomes, smart drug delivery systems against *Candida* species. J. Drug Deliv. Sci. Technol..

[13] Jaradat N., Abdallah S., Al-Maharik N., Altamimi M., Hawash M., Qneibi M., Abu Khair A., Zetawi A., Jabarin L. (2022). Constituents, Antibacterial Adhesion, Cytotoxic and in Vitro Metastasis Blocking Properties of *Salvia fruticosa* Essential Oils from Three Palestinian Localities. Chem. Biodivers..

[14] Sarac N., Ugur A. (2009). The in vitro antimicrobial activities of the essential oils of some Lamiaceae species from Turkey. J. Med. Food.

[15] Pitarokili D., Tzakou O., Loukis A., Harvala C. (2003). Volatile metabolites from *Salvia fruticosa* as antifungal agents in soilborne pathogens. J. Agric. Food Chem..

[16] Arumugam T., Ghazi T., Chuturgoon A.A. (2021). Molecular and epigenetic modes of Fumonisin B1 mediated toxicity and carcinogenesis and detoxification strategies. Crit. Rev. Toxicol..

[17] Bilia A.R., Piazzini V., Risaliti L., Vanti G., Casamonti M., Wang M., Bergonzi M.C. (2019). Nanocarriers: A Successful Tool to Increase Solubility, Stability and Optimise Bioefficacy of Natural Constituents. Curr. Med. Chem..

[18] Bilia A.R., Piazzini V., Guccione C., Risaliti L., Asprea M., Capecchi G., Bergonzi M.C. (2017). Improving on Nature: The Role of Nanomedicine in the Development of Clinical Natural Drugs. Planta Med..

[19] Angelico R. (2022). Micro/Nano Emulsions: Smart Colloids for Multiple Applications. Nanomaterials.

[20] Pavoni L., Perinelli D.R., Bonacucina G., Cespi M., Palmieri G.F. (2020). An Overview of Micro- and Nanoemulsions as Vehicles for Essential Oils: Formulation, Preparation and Stability. Nanomaterials.

[21] Battaloglu R. (2021). Calculation of Retention Indices of Essential Oils with the aid of the Van den Dool and Kratz equation and Bézier Curves. Authorea.

[22] Adams R.P. (1995). Identification of Essential Oil Components by Gas Chromatography/Quadrupole Mass Spectroscopy.

[23] Smail S.S., Ghareeb M.M., Omer H.K., Al-Kinani A.A., Alany R.G. (2021). Studies on Surfactants, Cosurfactants, and Oils for Prospective Use in Formulation of Ketorolac Tromethamine Ophthalmic Nanoemulsions. Pharmaceutic.

[24] Katona G., Sipos B., Ambrus R., Csóka I., Szabó-Révész P. (2022). Characterizing the Drug-Release Enhancement Effect of Surfactants on Megestrol-Acetate-Loaded Granules. Pharmaceuticals.

[25] Labrasol ALF, Gattefosse. https://www.gattefosse.com/pharmaceuticals/product-finder/labrasol-alf/.

[26] Vanti G., Grifoni L., Bergonzi M.C., Antiga E., Montefusco F., Caproni M., Bilia A.R. (2021). Development and optimisation of biopharmaceutical properties of a new microemulgel of cannabidiol for locally-acting dermatological delivery. Int. J. Pharm..

[27] Sacco C., Donato R., Zanella B., Pini G., Pettini L., Marino M.F., Rookmin A.D., Marvasi M. (2020). Mycotoxins and flours: Effect of type of crop, organic production, packaging type on the recovery of fungal genus and mycotoxins. Int. J. Food Microbiol..

[28] Sinclair R.G., Gerba C.P. (2011). Microbial contamination in kitchens and bathrooms of rural Cambodian village households. Lett. Appl. Microbiol..

[29] European Chemicals Agency, Performance Criteria Overview of (EN) Standards, Test Conditions, and Pass Criteria. https://echa.europa.eu/documents/10162/20733977/overview_of_standards_test_conditions_pass_criteria_en.pdf/f728e5c1-afd6-4c25-8cc3-ca300cd9b1cf.

[30] Skoula M., El Hilali I., Makris A.M. (1999). Evaluation of the genetic diversity of *Salvia fruticosa* Mill. clones using RAPD markers and comparison with the essential oil profiles. Biochem. Syst. Ecol..

[31] Karousou R., Vokou D., Kokkini S. (1998). Variation of *Salvia fruticosa* Essential Oils on the Island of Crete (Greece). Bot. Acta.

[32] Papageorgiou V., Gardeli C., Mallouchos A., Papaioannou M., Komaitis M. (2008). Variation of the chemical profile and antioxidant behavior of *Rosmarinus officinalis* L. and *Salvia fruticosa* Miller grown in Greece. J. Agric. Food Chem..

[33] Koliopoulos G., Pitarokili D., Kioulos E., Michaelakis A., Tzakou O. (2010). Chemical composition and larvicidal evaluation of *Mentha*, *Salvia*, and *Melissa* essential oils against the West Nile virus mosquito *Culex pipiens*. Parasitol. Res..

[34] Schmiderer C., Torres-Londoño P., Novak J. (2013). Proof of geographical origin of Albanian sage by essential oil analysis. Biochem. Syst. Ecol..

[35] FDA, GRAS Extemption Claim. https://www.fda.gov/files/food/published/GRAS-Notice-000622--Emulsified-fish-oil.pdf.

